# A dietary supplement to improve the quality of sleep: a randomized placebo controlled trial

**DOI:** 10.1186/1472-6882-10-29

**Published:** 2010-06-22

**Authors:** Catherine Cornu, Laurent Remontet, Florence Noel-Baron, Alain Nicolas, Nathalie Feugier-Favier, Pascal Roy, Bruno Claustrat, Mitra Saadatian-Elahi, Behrouz Kassaï

**Affiliations:** 1 INSERM, CIC201, Lyon, F-69000 France; CHU Lyon, Service de Pharmacologie Clinique, Lyon, F-69000 France; Univ Lyon, UMR 5558, Lyon, F-69000 France; 2 Unité d'Exploration Hypnologique, Service Hospitalo-Universitaire de Psychiatrie (Pr T. D'Amato ), Hôpital du Vinatier, 95 Bd Pinel, 69677 Bron - France; Univ Lyon, UMR 5167, Lyon, F-69000 France; 3 Hospices Civils de Lyon, Service de Biostatistique, Lyon, France; CNRS, UMR 5558, Villeurbanne, France; Université Claude Bernard, Laboratoire Biostatistique Santé, Lyon, F-69003, France; 4 Service d'hormonologie, Centre de médecine nucléaire, Hôpital Cardiologique, 28 avenue Doyen Lepine, 69500 BRON, France; INSERM U846, Lyon, France

## Abstract

**Background:**

To evaluate the effect of a dietary supplement containing polyunsaturated fatty acids, in association with *Humulus lupulus *extract, on the quality of sleep using the Leeds sleep evaluation questionnaire (LSEQ) in subjects with moderate to severe sleep disorders.

**Methods:**

Randomized placebo-controlled trial, in a Population-based setting. Participants were adult patients 25 to 65 years old with a chronic primary insomnia who volunteered for the study. The tested intervention consisted of two soft gelatine capsules per day, containing either the dietary supplement (active group) or olive oil (placebo group) for a month. Subjects could also volunteer for two ancillary studies on melatonin and actigraphy. Evaluation criteria included i) perception of the quality of sleep at the end of treatment using the LSEQ questionnaire, ii) sleep efficiency measured by one-week actigraphic movement measurement performed before and during the treatment in a subsample of subjects, iii) night melatonin and 6 sulfatoxymelatonin (aMT6S) urine rates in a subsample of subjects.

**Results:**

The average of Leeds score was similar in both groups (p = 0.95). A marked improvement in the quality of sleep was observed in both placebo (62%) and active (65%) group (p = 0.52). The evolution of urinary melatonin, aMT6S, and of the Mel/aMT6S ratio showed no differences between the two groups. Sleep efficiency, as measured by actigraphy, improved similarly in both groups during the treatment period, from 72% to 76% and 75% in the active and placebo group respectively (p = 0.91).

**Conclusions:**

The dietary supplement had neither effect on the perceived quality of sleep, nor on the melatonin metabolism and sleep-wake cycle.

Trial registration: clinical trials.gov:NCT00484497

## Background

The estimated incidence of primary insomnia in the general population is 10 to 20% [1-3]. The consequences of this disorder are an impaired quality of life, an increase in medical consultations and in hypnotic drug consumption [4]. Increase in drug consumption reinforces the risk of work accident and absenteeism, resulting in a decrease in productivity [1,2]. It can also leads to adverse cognitive and psychomotor effects, daytime sleepiness [5], loss of memory, increased risk of falling in old subjects [6], and impaired driving abilities [7]. Alternative therapeutics would therefore be welcome.

Melatonin, a hormone produced mostly during the night by the pineal gland, displays the role of an endogenous synchronizer, especially on the sleep-wake cycle [8]. Melatonin receptors are present at the level of the endogenous circadian clock located in the suprachiasmatic nuclei.

The study product is a dietary supplement composed of natural components. It contained a specific ratio of polyunsaturated fatty acids (PUFA, linolenic and linoleic acids), in association with *Humulus lupulus *extract. PUFA were claimed to favor melatonin synthesis [9]. The short half-life of melatonin requires the administration of high doses to balance its quick metabolism. Recently, a controlled release melatonin preparation has been shown effective in insomnia in aged patients [10-13]. *Humulus lupulus *extract has been shown to decrease the hepatic metabolism of drugs which are metabolised by the CYP1A2, a cytochrome that also allows melatonin transformation into its main metabolite: 6 sulfatoxymelatonin (aMT6s) [10]. This combined action could increase melatonin bioavailability and is likely to reset the endogenous biological clock. Besides, *Humulus lupulus *extract could directly act on sleep by enhancing the melatonin-mediated effects on the sleep-wake cycle [10].

Our objective was to evaluate the effect of the dietary supplement on the quality of sleep using the Leeds sleep evaluation questionnaire (LSEQ) in adult subjects with moderate to severe primary insomnia in a prospective double blind, randomized, placebo-controlled clinical trial.

## Methods

### Study population

Overall, 101 patients were enrolled between September 2006 and June 2007. The study was carried out at the Clinical Investigation Centre (CIC) located at the Lyon university hospital (France).

Subjects were invited to volunteer for the study through press announcements and a website for healthy volunteers' recruitment http://www.volterys.fr/. On the day of enrolment, subjects had a medical visit and a written consent was obtained after further information has been given.

The study was conducted in accordance with the Declaration of Helsinki. The protocol was approved by Lyon Ethics Committee (Comité de Protection des Personnes Lyon B) on July 18^th ^2006.

**Figure 1:**
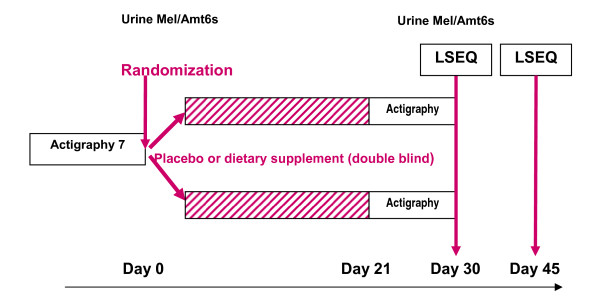
**Study design**.

The study design is shown in Figure 1. In addition to the main study, subjects could volunteer for two ancillary studies before randomization. The first ancillary study was based on overnight urinary melatonin and aMT6s measurements (melatonin sub-study) and the second, a one-week actigraphic movement measurement (Actigraphic sub-study).

Men and women aged 25 to 65 years, with a chronic primary insomnia were eligible to participate to the main study. Subjects had to be affiliated to the French Social Security System. Primary insomnia was defined according to the Diagnostic and Statistical Manual of Mental Disorders [14] (DSM-IV) and the International Classification of Sleep Disorders [15]. The severity of insomnia was classified by using the Insomnia Severity Index (ISI) [16]. The baseline minimum inclusion ISI score was 12, but was changed to strictly superior to 10 on February 6^th^, 2007, due to recruitment difficulties.

### Exclusion criteria

Sleep disorders secondary to another health problem; pharmacological resistance to common hypnotic drugs; any ongoing long term treatment (except contraceptive and postmenopausal hormone replacement); acute disease during the previous three months; medical history that could interfere with sleep; history of psychiatric disorder in the five years preceding the inclusion (detected using the MINI questionnaire) [17], depressive disorder measured by a score above 30 (or identification of a risk of suicide) on the Beck self-questionnaire [18], pregnant or lactating women; subjects for whom a poor adherence to the study protocol can be anticipated (i.e. likely to leave the study area); individuals protected by law; lifestyle habits which would modify the wake-sleep rhythm (e.g. night work), or which would be likely to be modified during the study period; known allergy to one of the dietary supplement components; subjects with excessive daytime sleepiness as measured by an Epworth score equal to or superior to ten [19].

### Randomization/intervention

A permuted-block algorithm was used for randomization. Concealed allocation was performed by the coordination centre after eligibility check and baseline data collection. Patients were asked to take two soft gelatine capsules per day two hours before sleep for a month. Capsules contained the dietary supplement (active group) or olive oil (placebo group). The dietary supplement is currently on the market under the brand name Cyclamax^® ^and contains 260 mg Soya oil (*Glycine max*), 173 mg.

Cade oil (*Cannabis sativa*), 50 mg Houblon (*Humulus lupulus*) and 6 mg Soya lecithin. Its envelope is composed of modified starch, glycerine, *Iota carrageenan*, glyceryl monostearate, titanium dioxide, lecithin certified non-GMO, and disodium phosphate.

### Evaluation criteria and follow-up

The primary criterion was to evaluate the perception of the quality of sleep at the end of the treatment (D30) using the LSEQ questionnaire [20]. This is a standardized self-reporting instrument comprising ten 100-mm visual analogue quetions that pertain to four items: i) the ease of getting to sleep (GTS, items 1-3), ii) the quality of sleep (QOS, items 4, 5), iii) awakening from sleep (AFS, items 6, 7) and iv) behaviour following wakefulness (BFW, items 8-10). The LSEQ is a sensitive indicator of subjectively felt changes in sleep latency, and sleep quality. For each question, the subject responds by placing a vertical mark on the line (0-100 mm) to indicate his/her present self-evaluation. The position of the mark indicates the nature and extent of the change, i.e. the middle of the line indicates no change, and the larger the changes the closer to the end of the line (improvement on the left, and impairement on the right). The score has been used in acute and chronic regimens. LSEQ was measured at day 30, and day 46 [11,12,21].

Secondary criteria included the LSEQ at day 46 (D46).

### Other measurements

A daily sleep diary was recorded by the participants to obtain information on total sleep duration, duration of first sleep (i.e. duration of sleep till the first awakening), number and duration of night wakes, number and duration of day sleep. Sleep efficiency (duration of sleep/time spent in bed) was then computed.

The patients' evolution was rated by the investigator, using the Clinical Global Impression at entry and the Clinical global impression change (from very much better to very much worse) between inclusion, D30 and D46. Safety was measured by recording adverse events.

Screening characteristics measured only at baseline included medical history,` the Insomnia Severity Index (ISI) questionnaires, a MINI questionnaire for psychiatric disorders screening, results, the Beck Depression Index 17, sleep characteristics, Epworth Sleepness Scale,), and the morningness-eveningness questionnaire of Horne and Ostberg which evaluates diurnal preference [16-19,22].

Follow-up included questionnaires on D30, evolution of symptoms and compliance to treatment (number of pills used assessed by pill count/number of study days). A follow-up visit was performed on D46 to assess a potential persisting treatment effect.

### Melatonin sub-study

The night melatonin and aMT6s urine rates were measured before and at the end of the treatment. Night urines were collected from 8:00 PM to 8:00 AM. Total urine volume was measured and a 5 ml-sample was frozen. Melatonin and aMT6s concentrations were determined using radio-immunoassays (RIA) [23,24]. Briefly, melatonin RIA was performed on urine extract using an I125tracer and a rabbit melatonin antibody. The aMT6s RIA assay was performed on diluted urine using an I125tracer and a rabbit melatonin antibody. Results were expressed in nano-gramme (ng) or pico-gramme (pg) per hour for aMT6s and melatonin respectively. Melatonin/aMT6s ratio was also determined.

#### Actigraphy sub-study

A one-week actigraphic movement measurement was performed twice, using Actigraph AW4 (actiwatchplus^®^). The corresponding software (activity and sleep analysis, 2001, Cambridge Neurotechnology Ltd, UK) allowed computing sleeping patterns, sleep efficiency, latency, fragmentation index, and average wake movement.

Patients had to wear the device around their non dominant wrist during the one week period before and while on treatment.

### Data collection

Data were collected on Case Report Forms by the investigators, and entered in a data base using the Clininfo SA software (Clininfo SA, 99 rue de Gerland, 69007 Lyon, France).

#### Sample size

The sample size was calculated according to data given by Hindmarch [25].

Using a between-group mean score difference of 10 points, a score standard deviation of 15 points, α = 5%, power = 90%, the number of subjects needed is 100; i.e., 50 subjects per group.

#### Statistical Analysis

The main outcome measure comprises four scores relative to dimension GTS, QOS, AFS, and BFW measured in each patient. Using a mixed model, we studied the effect of the treatment on these scores considering them as repeated measurements in each subject [26]. The objective was to compare the mean of each score in the control group to the corresponding mean in the treatment group, taking into account the high correlation between the four scores. For example, an individual with a high QOS would probably have a high BFW too. This correlation should be taken into account to allow statistical inference about the means of theses variables.

When analysing such repeated measurements or longitudinal data, the mixed model is the method of reference because it models explicitly the correlation between the outcome variables and provides correct statistical inference. Furthermore, this method allows a simultaneous testing of the effects of the treatment on the four dimensions through a single test with an alpha risk of 5% (Additional file 1). The effect of the treatment on sleep efficiency was also assessed using a mixed model.

The effect on other variables of interest was tested using Student's test for all continuous variables and Fisher's exact test for categorical variable.

## Results

**Figure 2:**
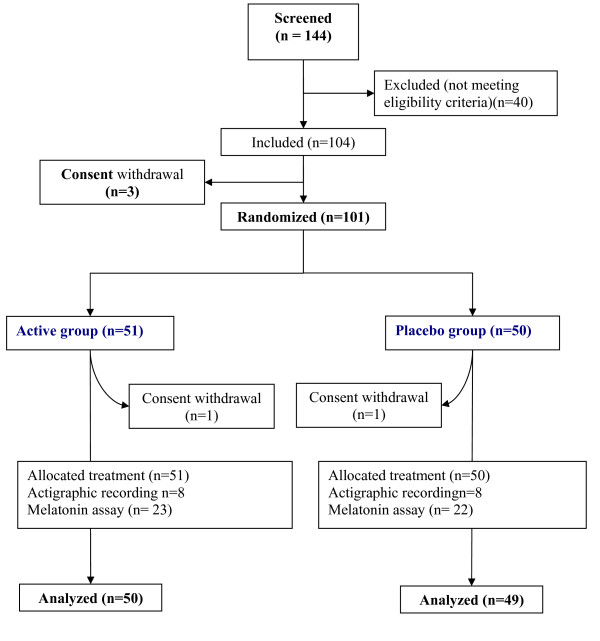
**Flow diagram of participants**.

Overall, 101 subjects were enrolled and randomly assigned to the active dietary supplement or placebo. The flow diagram and baseline characteristics of the study population are shown in Figure 2 and Table 1 respectively. Of the 101 patients randomized, 51 were in the active group (19 men and 32 women) and 50 (13 men and 37 women) in the placebo group. The mean age was 41 years (range 25-64). Mean body weight, smoking habits, socio-professional status and diurnal preference did not differ between the groups. In both groups, 33% had subliminal, 55% had moderate, and 12% had severe insomnia. Compliance with treatment were 98.3 (SD 10.5) and 97.5 (SD 8.1) in placebo and active group respectively.

**Table 1:** Baseline characteristics of the study subjects

	Active group (n = 51)	Placebo group (n = 50)
**Male (%)**	19 (37)	13 (26)

**Age (mean, range)**	40,9 (25,1-64,7)	41,8 (24,9-64,8)
***Patient < 40 years***	23 (45%)	24 (48%)
***Patient > 40 years***	28 (55%)	26 (52%)

**Mean body weight, kg (range)**	67 (46-103)	65,4 (45-99)

**Non smokers (%)**	35 (69)	32 (64)

**Socio-professional status**		
Intermediate	18	17
Students	11	08
Superior	10	11
Employee	9	13
Retired	3	1

**Morningness-eveningness**		
*Quite morning type*	2	3
*Rather morning type*	13	14
*Neither morning, nor evening type*	23	23
*Rather evening type*	9	6
*Quite evening type*	4	4

**ISI Score**		
*Score 8 to 14 (Minor insomnia)*	17 (33%)	17 (33%)
*Score 15 to 21 (Moderate Insomnia)*	28 (55%)	28 (55%)
*Score 22 to 28 (Severe insomnia)*	6 (12%)	5(12%)

ISI score stands for Insomnia Severity index.

Quality of sleep, as assessed by the LSEQ, was available for 99 patients (2 missing data, one in each group). The average of Leeds score was similar in both groups (Likelihood Ratio Test = 0.71, p = 0.95): mean GTS 173.9 and 170.5, QOS 124.8 and 120.1, AFS 161.6 and 159.1, and BFW 118.6 and 118.1 in the placebo group and in the active group respectively (Table 2). The dietary supplement did not improve sleep compared to the placebo condition. The results remained unchanged after using two methods of missing data replacement, the maximum bias method, and the mean value of the group.

**Table 2:** Primary criterion, D 30-Leeds score

	Active Group (n = 50)		Placebo Group(n = 49)	
	Mean (SD)	95% CI	Mean (SD)	95% CI
**GTS**	170.5 (30.7)	161.8; 179.2	173.9 (51.3)	159.2; 188.7
**QOS**	120.1 (31.4)	111.2; 129	124.8 (41)	113; 136.6
**AFS**	159.1 (42.6)	147;171.2	161.6 (46.7)	148.2; 175
**BFW**	118.1 (31.2)	109.2;126.9	118.6 (39.5)	107.3; 129.9

Mean, SD (standard deviation), and 95% confidence interval for GTS (getting to sleep), QOS (quality of sleep), AFS (awaking from sleep), and BFW (behaviour following wake) scores in both groups.

**Figure 3:**
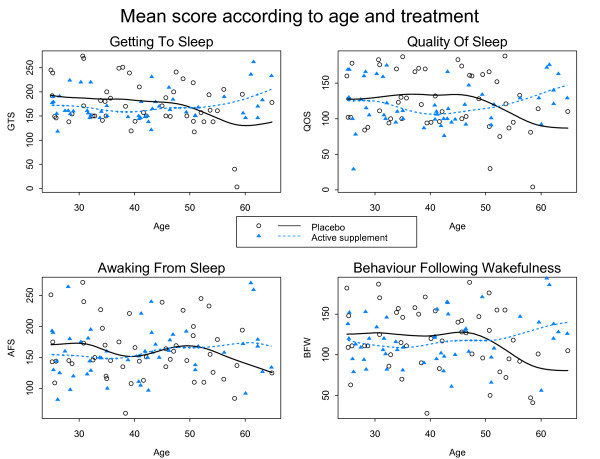
**Mean score (solid curves and dashed curves) according to age and treatment group for GTS, QOS, AFS and BFW: observed score for each patient are also plotted**.

A marked improvement in the quality of sleep was observed in both groups. We observed correlation between the four scores (r = 0.52 to 0.79). The high correlation between QOS and BFW (r = 0.755) suggested a good refreshing sleep. Figure 3 shows the mean of the scores according to the treatment group and the age of participants. The apparent divergence of the curves for very old ages might be due to very old subjects considered as outliers: three in the active group have a very good score, and two in the placebo group have a very bad score. There seems to be no clear relationship between score and age.

### Secondary criteria analyses

Secondary results are shown in the table 3. The D46 analysis was not performed because a persisting treatment effect could not be expected in the absence of effect at D30.

**Table 3:** Secondary criteria: sleep efficiency computed from the sleep diary

	Active group (n = 51)	Placebo group (n = 50)
	Mean (SD)	
**Sleep efficiency (%)**		
***Week 1***	80.9 (13.8)	81.5 (16.6)
***Week 4***	82.8 (12.7)	85.1 (13.2)
**Number of napping episodes**		
***Week 1***	0.13	0.11
***Week 4***	0.13	0.17
**General Clinical Impression**	N (%)	
***Worsening***	1 (2%)	3 (6%)
***Unchanged***	18 (36%)	14 (29%)
***Improved***	31 (62%)	32 (65%)

Mean, SD (standard deviation), and 95% confidence interval in both groups.

The evolution of sleep diary data, i.e. sleep efficiency, total sleep duration, duration of first sleep, number and duration of night awakenings, number and duration of nap episodes did not differ between groups.

The investigator Clinical Global Impression change showed an improvement in 62% of patients in the placebo group and in 65% of those in the active group (p = 0.52).

Twenty two (44%) patients in the placebo group and seventeen (33.3%) in the active group had minor adverse event represented mainly (55%) by headache in both groups.

#### Melatonin sub-study

The evolution of urinary melatonin, aMT6S, and of the Mel/aMT6S ratio showed no differences between the two groups (all Ps > 0.05, Table 4).

**Table 4:** Evolution of urinary melatonin, 6 sulfatoxymelatonin, and melatonin/6 sulfatoxymelatonin ratio

	Active group			Placebo group		
		
	Baseline	End of Study	Difference	Baseline	End of Study	Difference
Melatonin (ng/h),	8.1 (5.9)	10.3 (11.5)	+2.2 (11.3)	8.0 (5.4)	7.3 (5.2)	-0.7 (5.0)
aMT6S (ng/h)	362.9 (256.0)	333.4 (206.2)	-29.4 (138.2)	291.0 (144.4)	294.3 (144.5)	+3.3 (111.2)
Mel/aMT6S*	0,0301 (0,0329)	0,0339 (0,0327)	+0,004 (0,037)	0,0301 (0,0201)	0,0319 (0,0327)	+0,002 (0,030)

Mean (standard deviation)

* mean of all patients ratios

#### Actigraphic sub-study

Actigraphic data were available in 54 patients out of 61 included. One patient declined to participate and one of the recordings was not available for five patients. Sleep efficiency improved similarly in both groups during the treatment period, from 72% to 76% and 75% in the active and placebo group respectively (p = 0.91).

## Discussion

The present study showed that the tested dietary supplement did not improve the quality or the duration of sleep as compared to the olive oil placebo capsules. Furthermore, no significant change of melatonin or aMT6S urinary excretion was observed, suggesting that the dietary supplement had no effect on the melatonin metabolism.

Potential limitations of the study are first the type of placebo used. The placebo capsules contained olive oil. However, it is unlikely that olive oil could have any activity to improve sleep. No bibliographic references with both sleep disorders and olive oil have been found. In their meta-analysis, Mc Call et al. observed a mean diminution of subjective sleep latency of 13 min, a mean diminution of polysomnographic latency of 2.5 min, a mean increase in the total sleep duration of 13 min [27]. These figures are close to those observed in our placebo group: 15 min and 9 min reduction in the subjective sleep latency in actigraphic and diary measurements, an 11.5 and a 17 min increase in the total sleep duration measured by actigraphy and by the sleep diary respectively. This suggests that our placebo group was a true placebo group. It is well known that cognitive and compartmental therapies are effective in mild insomnia. In the present study, patients had a close follow-up, and were asked to record a sleep diary. The type of care and follow-up given during a clinical trial is likely to improve sleep in mild insomnia, and is an extra justification of the need for placebo-controlled trials.

The LSEQ questionnaire allowed to measure subjective ratings of the drug effects on the quality of sleep, early morning behaviour, and both direction and magnitude of behavioural changes by using a series of 10 cm line analogue scales. Although the questionnaire was first constructed to assess the change after one night, it has been extensively used for assessing drugs. Zisapel and col. [21] identified 83 studies that reported the use of the LSEQ for psychopharmacological investigations of drug effects on self-reported aspects of sleeping in studies involving a variety of psychoactive agents in healthy volunteers, depressed and insomnia patients. The authors concluded that the LSEQ is a robust and reliable instrument for psychopharmacological evaluations and can provide consistent and meaningful measures for estimating the effectiveness of sleep modulators and sedative-hypnotic drugs with various drug regimens, from one day to 24 week-treatment durations [21].

Finding mild therapeutic agents to improve sleep and to avoid hypnotic drug consumption would be very useful. Very few studies with adequate methodology have been performed to assess the impact of dietary supplement therapies on sleep improvement. In a meta-analysis including 16 randomised trials and 1093 patients and summarizing evidence of the effect of valerian, sleep was improved in significantly more patients (RR: 1.8, 95% CI = 1.2-2.9), without side effects [28]. However, the authors found many methodological problems, publication bias, and considerable variation in valerian doses and preparations, and treatment duration that led them to conclude the need for further studies. Melatonin has also shown clinically meaningful improvements in sleep quality, morning alertness, sleep onset latency and quality of life in primary insomnia patients aged over 55 years [11,12]. A fixed combination of valerian and hops was shown to favourably influence sleep in a similar way as melatonin [29,30].

The present study was adequately sized and well conducted, since there are very few missing data. It included clinical and explanatory endpoints, such as melatonin/aMT6S ratio and actigraphic measurement of sleep parameters. The study targeted middle age (25-65 years) subjects with moderate to severe insomnia, who had never been treated regularly. The age limit was fixed at 25 years, since in younger patients, approximately 10% of patients with primary insomnia may have delayed sleep phase syndrome which is sensitive to melatonin treatment [31].

Our results cannot be extended to other types of population, e.g. elderly patients who might benefit from such supplements. Clinical trials specifically designed for these objectives are warranted.

Recommendations for prescribing hypnotic drugs in primary insomnia are to advise short period (one week) use of drugs only if cognitive behavioural therapy is not effective [32]. However, long-term treatment of insomnia is sometimes needed. Hypnotic drugs currently available remain unsatisfying in many cases, because of their insufficient efficacy and excess of side effects.

## Conclusions

The dietary supplement was not found effective in any of the tested outcomes. New and emerging anti-insomnia agents may prove useful in the long-term treatment of chronic insomnia. Further research is needed to establish the benefit of such treatments.

## Competing interests

The study was funded by PERSEE MEDICA, the manufacturer of the product.

## Authors' contributions

CC, FNB, BK conceived the study, participated in its design and coordination, drafted and reviewed the manuscript. LR performed the statistical analysis. CC, FNB, NFF, AN, BK recruited patients and PR supervised the statistical analysis. All authors read and approved the final manuscript. Hormone assays were performed in BC's laboratory

## Pre-publication history

The pre-publication history for this paper can be accessed here:

http://www.biomedcentral.com/1472-6882/10/29/prepub

## Supplementary Material

Additional file 1 **Description of mixed model**. The file describes in details the mixed model used in the study to test simultaneously four scores relative to dimension GTS, QOS, AFS, and BFW measured in each patient Click here for file
