# Supplementary material for: A dietary supplement to improve the quality of sleep: a randomized placebo controlled trial
Source: BMC Complement Altern Med. 2010 Jun 22;10:29. doi: 10.1186/1472-6882-10-29 (PMC2901361; doi:10.1186/1472-6882-10-29)
Supplement: Additional file 1 — Description of mixed model. The file describes in details the mixed model used in the study to test simultaneously four scores relative to dimension GTS, QOS, AFS, and BFW measured in each patient [file 1472-6882-10-29-S1.DOC]

**Additional file 1:** Description of mixed model

Let Y_ij_ be the score of patient i (i=1, …, n) in a dimension j (j=GTS, QOS, AFS, or BFW). The model may then be written as follows:

Y_ij_=  β_1_I_gts,i,j_ + β_2_I_qos,i,j_ + β_3_I_afs,i,j_ + β_4_I_bfw,i,j_ + β_5_I_gts*tt,i,j_ + β_6_I_qos*tt,i,j_ + β_7_I_afs*tt,i,j_ + β_8_I_bfw*tt,i,j_ +

u_i1_I_gts,i,j_ + u_i2_I_qos,i,j_ + u_i3_I_afs,i,j_ + u_i4_I_bfw,i,j_ +e_ij_

The parameters to estimate are the βs as well as the variances and covariances of the random effects u_i1_, u_i2_, u_i3_ et u_i4_ that provides the correlation between the scores. For example, cov(u_i2_;u_i4_) measures the correlation between QOS and BFW. I is an indicator variable:

I_gts,i,j_ =1 when observation j relative to patient i is GTS-related and =0 otherwise.

I_gts*tt,i,j_ =1 when observation j relative to patient i is GTS-related and patient i treated by cyclamax, and =0 otherwise.

Using this parameterization, β_5_, β_6_, β_7_, β_8_ represent, respectively, the differences in the mean GTS, QOS, AFS, and BFW scores between the test and the control group, respectively.

The main hypothesis H0 “none of the four dimensions is different in average between tests and controls” may then be tested through the simultaneous testing of the 4 parameters β_5_, β_6_, β_7_, β_8_ against zero. This may be done using the Likelihood Ratio Test that compares the likelihood of a model that includes terms relative to β_5_, β_6_, β_7_, β_8_ to the likelihood of a model that does not include these terms (this difference of likelihood follows a chi-square distribution with 4 df).
